# Analysis of the Quality and Chemical Composition of Double-Yolked Eggs Compared to Those of a Normal Structure

**DOI:** 10.3390/ani14111568

**Published:** 2024-05-25

**Authors:** Kamil Drabik, Karolina Wengerska, Kornel Kasperek, Sebastian Knaga, Justyna Batkowska

**Affiliations:** 1 Institute of Biological Basis of Animal Production, University of Life Sciences in Lublin, 13 Akademicka St., 20-950 Lublin, Poland; kamil.drabik@up.lublin.pl (K.D.); kornel.kasperek@up.lublin.pl (K.K.); justyna.batkowska@up.lublin.pl (J.B.); 2 Department of Animal Biotechnology and Genetics, Bydgoszcz University of Science and Technology, 28 Mazowiecka St., 85-084 Bydgoszcz, Poland; sebastian.knaga@pbs.edu.pl

**Keywords:** table eggs, chemical composition, fatty acid profile, egg defects

## Abstract

**Simple Summary:**

Simple Summary: Double-yolked eggs occur in reproductive flocks, especially at the beginning of the laying period. Although the reasons for their occurrence are fairly well understood and work is underway to reduce them, the problem still affects about 1–2% of chicken eggs. Due to reduced hatchability, such eggs are hatchery waste, but may be of interest in the context of consumer eggs. The present study aimed to analyse the quality and technological suitability of eggs with a double yolk compared to eggs with a normal structure. Differences were shown in the proportion of individual egg morphological elements in the weight of the whole egg, as well as in the chemical composition. Small but positive differences in the content of unsaturated fatty acids suggest the possible use of double-yolked eggs as table eggs.

**Abstract:**

The study material consisted of 360 eggs from a reproductive flock of meat-type hens; 240 were double-yolked eggs and 120 were single-yolked as a control group. The eggs were numbered individually and then analysed for their quality in terms of characteristics of the whole egg (weight, shape index, specific gravity), shell (colour, strength, weight, density), albumen (pH, height, weight, Haugh units) and yolk (colour, weight, shape index, pH). During the analyses, yolks were sampled for analyses including basic composition, fatty acid profile (by gas chromatography) and fatty acid indices. It was found that double-yolked eggs differed significantly from single-yolked ones in terms of weight, proportion of individual elements in the egg weight, total protein content in the yolks as well as in terms of the fatty acid profile and their indices both due to the presence or absence of two yolks and in the context of the individual yolks analysed. The results indicate the possibility of using double-yolked eggs as table eggs due to the absence of negative effects stemming from being double-yolked and the increased content of biologically important components such as fatty acids.

## 1. Introduction

The occurrence of a double yolk is one of the most common abnormalities of the egg’s internal structure. It is estimated that in the case of meat-type hens, their occurrence at the beginning of laying is 5–12% [1], while throughout the whole laying cycle, double-yolked eggs account for only 1–2%. In the available literature, two basic mechanisms for the formation of double-yolked eggs have been described. In the first, two ova are released simultaneously during ovulation, with one ovulating at the normal time and the other ovulating prematurely. In the second, a yolk sac can retract in the oviduct as a result of stress with the simultaneous release of another yolk, such that both are surrounded by the same albumen, membranes and shell [2]. According to Salamon and Kent [3], there are three types of double-yolked eggs: the first type (A) is characterised by two yolks with separate vitelline membranes enclosed in a common chalaziferous albumen; in the second type (B), the two yolks have separate chalaziferous albumen but share common dense albumen; and in the third type 3rd (C), there is a double egg within a single shell [4,5]. In addition to the types of double-yolked eggs mentioned above, the occurrence of eggs with a double yolk and a shared vitelline membrane has also been observed. An extreme example of the occurrence of double-yolked eggs is the so-called egg-in-egg, where, as a result of stress, an ovum that the enveloping membranes have not completely surrounded is retracted in the oviduct and is absorbed by another ovum, with which it is enclosed in a single shell.

A number of factors may influence the formation of double-yolked eggs, from the age and type of birds, to their rearing system and nutrition [3]. Due to the fact that up to 25% of follicles in the early laying period develop in pairs, there is a chance of their simultaneous ovulation [6]. This process may be related to the gonadotropin-releasing hormone receptor, which is responsible for reproductive activity. In contrast, neuropeptide Y, which has a significant effect on the rate of egg production, is responsible for the release of the gonadotropin-releasing hormone itself [7]. The most frequently double-yolked eggs occur in the first several weeks of laying, when the hen’s endocrine system is not fully stabilised and ovarian follicles are developing in the still-maturing gonads. As reported by Zuidhof et al. [8], the ovaries are so mature in hens after 40 weeks of age that the follicles very rarely ovulate in pairs, resulting in the laying of eggs with a single yolk. Despite the multitude of factors that may influence the occurrence of two-yolk eggs, their heritability compared to the occurrence of other defects is relatively high [9]. Abplanalp et al. [10] attempted to select White Leghorn laying lines laying double-yolked eggs. Initially, such eggs accounted for 23% to 55% of all eggs laid, but after the 25th week, the frequency of double-yolked eggs decreased.

Double-yolked eggs, due to the belief that they have a higher nutritional value, are extremely popular among consumers. In Asian countries, they are often considered a symbol of good fortune, and their market price is much higher than single-yolked [11]. However, for producers, these eggs usually represent a kind of waste, due to non-standard size that does not fit in traditional egg boxes and often reduced shell strength, which generates losses. That is why they are commonly sold directly from the farm to avoid transportation inconveniences and loses. Due to the fact that double-yolked eggs may form in various oviposition stages, it was also hypothesised that the yolks may slightly differ from each other. Therefore, this study aimed to analyse the quality and technological usefulness of double-yolked eggs in comparison with eggs of a normal structure.

## 2. Materials and Methods

The material for the study consisted of 360 chicken eggs obtained from the ROSS 308 (Aviagen^®^, Huntsville, AL, USA) chicken broiler reproduction farm. The eggs were obtained from birds aged 29 weeks. All eggs were collected on the same day and the presence of a single or double yolk was verified by candling. The material thus obtained was divided into two groups: a control group consisting of normal eggs (*n* = 120) and a test group composed of double-yolked ones (*n* = 240). All eggs were individually numbered before proceeding to further analyses.

### 2.1. Eggs’ Quality Evaluation

All collected eggs were subjected to a quality analysis taking into account the characteristics of the whole egg as well as its particular elements. The EQM analysis kit (Egg Quality Measurement, TSS^®^, York, UK) was used for the analysis. The following characteristics of the whole egg were analysed: shape index (determined from the ratio of the short and long axis of the egg using an electronic caliper), weight (using an electronic scale with an accuracy of 0.01 g) and specific gravity (determined based on the dry and wet weight of the egg according to Archimedes’ principle). Based on the weight of the particular morphological elements of the egg, their proportion in the egg weight was calculated.

Shell characteristics were registered as follow: strength (the force required to break the shell, using an Instron Mini 55), colour (as a percentage of reflected light), thickness (measured at the equator using a micrometre screw), density (calculated from the surface area and volume of the shell [12]) and the cohesiveness. 

The parameters of the albumen quality assessed were its height (measured in the dense fraction of albumen using a sensor as part of the EQM kit), Haugh units [13] and pH (using a pH-meter with a glass electrode, C-401, Elmetron, Zabrze, Poland).

With regard to the egg yolk, its characteristics were determined, such as its weight (using an electronic balance with an accuracy of 0.01 g), colour (using the Roche 16-point scale, DSM^®^, Heerlen, The Netherlands), shape index (expressed as the ratio between the diameter of the yolk and its height measured using an electronic caliper) and pH (using a pH-meter with a glass electrode, C-401, Elmetron).

In double-yolked eggs, a visual assessment of the double-yolked type (Table 1) was made according to the methodology proposed by Romanoff and Romanoff [14]. Also, in these eggs, each yolk was analysed individually. The yolks collected separately by qualitative analysis were preserved and destined for further determinations. 

### 2.2. Basic Chemical Composition of Egg Yolks

A total of 30 yolks from eggs with a normal structure and 60 yolks from double-yolked eggs were allocated for analysis, with pairs of yolks taken from the same egg being analysed. The following parameters of the yolks basic composition were determined: dry matter content—samples of yolks of known weight were subjected to drying in a laboratory dryer (MEMMERT, Schwabach, Germany) at 105 °C until the weight in two measurements differed by no more than 0.004 g; and total nitrogen content—by Kjeldahl method using the KJELTEC™ 2300 automated system (FOSS, Hamburg, Sweden). From the result obtained, the crude protein content was determined by a calculation method multiplied by 6.25, while crude fat content was determined using the Soxhlet method. Hydrolysis was carried out using a SoxCap 2047 (FOSS, Sweden) and analysis was performed using a Soxtec 2050 fat analyser (FOSS, Sweden); crude ash content was determined after combustion of the samples in a muffle furnace at 550 °C.

### 2.3. The Fatty Acids Profile of Egg Yolks

The collected yolks were subjected to fat extraction using the Soxhlet method. Then, methyl esters were formed by trans-esterification with methanolic potassium hydroxide. Analysis of the proportion of particular fatty acids was performed by gas chromatography using a gas chromatograph (Varian GC, Palo Alto, GA, USA) with a flame ionisation detector with flame ionization detector (FID). Temperatures of injector and detector were 250 °C and 300 °C, respectively. The column temperature after injection was programmed to increase to 200 °C for 10 min, subsequently increased to 240 °C at the rate of 3 °C min^−1^. Then, the column temperature was held at the final temperature for 4 min. The carrier gas used for the analyses was helium (3 mL min^−1^). The methyl ester peaks of the sample were identified from the chromatogram according to European Union (EU) Regulations (method for the preparation and analysis of methyl esters of fatty acids. Method: EE 2568/91).

Based on the resulting proportions of specific fatty acids or groups of them, the values of selected indices were determined as follow:

- PI (Peroxidizability Index)—determining the susceptibility to lipid peroxide group formation [15]: PI = (% C_X:1_ × 0.025) + (%C_X:2_ × 1) + (%C_X:3_ × 2) + (%C_X:4_ × 4) + (%C_X:5_ × 6) + (%C_X:6_ × 8);
- AI (Atherogenicity Index)—determining the nutritional value of fat and its atherogenic properties [16]: AI = (C_12:0_ + 4 C_14:0_ + C_16:0_)/[ΣMUFA+ (ΣPUFA n6 + n3)];
- TI (Thrombogenic Index)—to determine the quality of fat and its effect on blood clot formation [16]: TI = (C_14:0_ + C_16:0_ + C_18:0_)/[0.5 × ΣMUFA + 0.5 × ΣPUFA(n6) + 3 × ΣPUFA(n3) + (n3/n6)];
- DFA—desirable fatty acids described as unsaturated [17]: DFA = MUFA + PUFA + C_18:0_;
- HFSA—hypercholesterolaemic saturated fatty acids [18]: HFSA = C_12:0_ + C_14:0_ + C_16:0_;
- h/H—ratio of acids showing hypocholesterolaemic/hypercholesterolaemic character [19]: Σ (C18:1 c9, C18:2 n-6, C18:3 n-3, C18:3 n-6, C20:2 n-6, C20:3 n-6)/Σ (C14:0, C16:0).

### 2.4. Statistical Data Analysis

The data obtained were statistically processed using the SPSS 24.0 statistical package [20]. The normality of the distribution of particular characteristics was analysed using the Shapiro–Wilk test. The frequencies of double-yolked eggs were verified using non-parametric χ^2^ test. For qualitative parameters, the Student’s *t*-test was used. For chemical composition, a one-factor analysis of variance (ANOVA) with Tukey’s multiple comparisons test was used. A significance level of *p* ≤ 0.05 was adopted.

## 3. Results

### 3.1. Egg Quality

**Figure 1:**
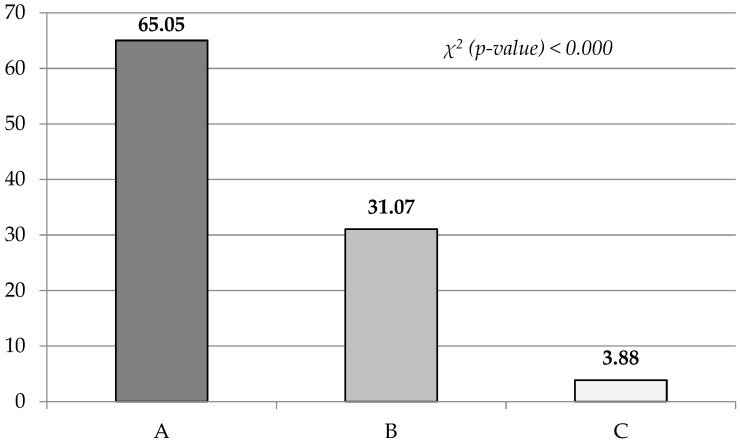
The type of double yolk (%) according to Romanoff and Romanoff [14].

During the analysis of egg quality, a classification of double-yolked eggs was conducted into specific types (Figure 1). It was observed that the highest proportion was attributed to eggs of type A, with the lowest count recorded for eggs of type C. 

Depending on the egg group, there were significant differences in the whole egg quality characteristics (Table 2). An increase in the length of the long axis was observed for double-yolked eggs (DY). Furthermore, they were characterised by significantly higher mass and specific gravity compared to eggs with normal structure. Due to the presence of two yolks, DY eggs demonstrated a higher yolk content and a decreased portion of albumen compared to single-yolked (SY) ones. However, no influence of being double-yolked on the shell proportion in the total egg weight was observed.

Regarding shell quality (Table 3), it was found that the presence of two yolks in DY eggs significantly increased the shell mass while simultaneously reducing its cohesion. No differences were noticed in other shell characteristics.

The most differences were observed between groups in the case of egg content quality (Table 4). Double-yolked eggs were characterised by considerably lower albumen height and a correlated Haugh unit value; however, the albumen weight was bigger. Moreover, the albumen in DY eggs had a higher pH compared to SY ones, which could indirectly affect other quality traits. In terms of yolk, only the pH of this component did not significantly differ between the studied groups. Concerning yolk weight, significantly lower value of this trait was found in SY eggs, though it should be noted that both yolks were weighed in DY eggs, significantly influencing the obtained results. Additionally, in SY eggs, egg yolks had higher colour values compared to the DY group data.

### 3.2. Basic Chemical Composition of Egg Yolks

Analysis of the basic composition of egg yolks (Table 5) revealed significant differences in total protein content, considerably higher in DY egg yolk levels. No significant differences were found for other items. No differences were found between particular yolks analysed within the DY group.

### 3.3. The Fatty Acids Profile of Egg Yolks

Analysis of fatty acid profiles (Table 6) showed differences between groups and particular yolks within the DY group. For saturated fatty acids, SY egg yolks had the highest content of myristic acid (C14:0) compared to DY ones. Different trends were observed for stearic acid (C18:0), eicosanoic acid (C20:0) and lignoceric acid (C24:0), with the lowest proportion in SY and the highest in DY egg yolks. More importantly, the proportion of these acids also differed significantly when comparing yolks within the DY group.

Significant differences between groups were also observed for monounsaturated fatty acids (MUFA). Myristoleic acid (C14:1) and palmitoleic acid (C16:1) content were significantly higher in SY egg yolks. DY eggs had the highest levels of oleic acid (C18:1) and cetoleic acid (C20:1), with no differences between particular yolks within this group. Erucic acid (C22:1) content was lowest in DY group A yolks, while yolks of group B and SY eggs did not significantly differ from each other and had significantly higher levels of this acid. No significant differences were found in polyunsaturated fatty acids (PUFA), although it is noteworthy that dihomo-γ-linolenic acid (C20:3 n6) and docosadienoic acid (C22:2 n6) were present in trace amounts in SY egg yolks, while their proportions could be determined in DY egg yolks. Based on the proportion of particular fatty acids, their indexes were estimated (Table 7). It was found that significant differences were determined only in n9 fatty acids. No differences were observed in terms of important raw material stability (PI) and pro-health values (AI, TI) of fatty acid indexes.

## 4. Discussion

Egg quality is influenced by a number of factors, but the impact of a defect such as being double-yolked has so far been poorly described in terms of egg usefulness or egg quality parameters. In our study, significant differences were found in egg weight, shape index and proportion of particular elements. This is a natural phenomenon due to the presence of an additional yolk. In the context of mass and shape, it has so far been analysed with a view to the possibility of increasing the detection level of double-yolked eggs at the hatchery stage. This has primarily used mass-based detection systems and/or imaging supported by appropriate mathematical algorithms [11,21]. This is the most reasonable approach due to the significantly reduced hatchability from double-yolked eggs. These egg characteristics and hatchability are influenced by physiological factors along with the age of the laying hen or its genotype [22] as well as those related to the housing system [23] or nutrition [24]. In our study, these variables were fully restricted, as a flock of the same age, genetically standardised, as well as fed the same feed mixtures. It must therefore be assumed that the observed differences were solely due to the occurrence of double yolk.

Increased egg weight is a physical load on the young laying hen’s body that can lead to increased mortality. On the other hand, the weight of available on-the-market table eggs is one of the main determinants of consumers’ purchasing choices [25]. Given the reduced hatchability value of such eggs, it can therefore be assumed that there is an opportunity to utilise them for consumption.

In our study, differences were also found in the quality parameters of particular morphological egg elements. The increased weight of the shells in double-yolked eggs is a result of their higher weight. In the case of shell cohesiveness, these differences can be directly attributed to the shell structure. Shell quality is an important aspect of egg quality as it prevents losses of raw material at the collection and subsequent distribution stages. A large number of factors shaping the quality of this element have been described in the available literature [26], but for our research, the vast majority have been standardised. On the other hand, as reported by Roberts [27], as birds age, egg weight increases, which is not accompanied by a proportional increase in shell weight. Although the study was conducted on a young flock, the occurrence of a double yolk significantly increased egg weight, which in turn explains the reduced cohesiveness of DY eggshells.

Variation was also observed in the context of egg content. An obvious one is the difference in yolk weight, which, in the case of double-yolked eggs, was treated as a single element. The significant differences in the number of Haugh units can be explained by the very method of their calculation based on egg weight and the height of the albumen dense fraction. Due to the higher egg weight, this result will automatically be slightly lower. An additional element affecting the height of the albumen dense is also the pH of the element. Raising the pH with time loosens the bonds that stabilise the gel structure of the albumen, influencing a reduction in its height [28]. The observed significantly higher pH in double-yolked eggs may therefore be an additional explanation for the difference in Haugh units.

Yolk is the most nutritionally valuable element of the egg, which furthermore has a significant impact on consumer acceptability. Although egg consumer preferences are highly regionalised, in general, consumers prefer yolks with more intense colour [25]. The eggs constituting the test material came from a reproductive flock where yolk colouration is of marginal importance, so the results obtained remain relatively low. It should be noted at this point that yolk colour is related to pigment content, mainly carotenoids [29], and can be controlled by appropriate nutritional supplements [30]. In the context of our study, it seems that this difference is solely due to the occurrence of the defect and may be related to the premature ovulation of the second yolk sphere. Perhaps due to their strong antioxidant properties, these pigments are deposited at the final stage of yolk maturation, but this hypothesis requires further research.

Although the egg is a generative cell and it is relatively difficult to make substitutions in its basic composition, significant differences in total protein levels have been found. In this context, the vast majority of studies point to the influence of nutritional additives, especially amino acids in various forms, such as L-lysine [31] or sulphur amino acids in general [32]. In the case of our study, similar factors were eliminated, so that the variability was generated solely by double-yolked status, but the reasons for this require further research.

The profile of fatty acids and the values of their indices are one of the most frequently addressed aspects of the chemical composition of table eggs. Their modification is most often performed by the use of additives containing significant amounts of unsaturated fatty acids such as vegetable oils [19]. In our study, the variability observed was mainly related to saturated and monounsaturated acids. Importantly, being double-yolked significantly affected the percentage of many acids such as stearic, myristic and margaric acids. At the same time, it should be noted that this variation mainly concerned acids with a high carbon chain. An interesting aspect is the variation in the proportion of oleic and eicosenoic acids also in yolks from the same egg. For the most part, similar observations were made for unsaturated acids and, therefore, those more susceptible to oxidative processes [33].

Analysis of the fatty acid indices showed changes in the content of n9 and desirable fatty acids. In the context of nutrition, this is an important issue due to the low intake of unsaturated acids. Although these issues are relatively frequently addressed in the literature, they are almost exclusively concerned with the use of nutritional supplements [34], whereas the physiological mechanisms of deposition of particular fatty acids require further research.

## 5. Conclusions

It was shown that the shells of double-yolked eggs, in contrast to single-yolked ones, are characterised by being heavier but with less cohesiveness due to the size of the eggs. In addition, double-yolked eggs were found to have a higher weight than SY eggs. Due to the presence of two yolks in the egg, the yolk mass of DY eggs is significantly higher, which also affects the lower yolk pigmentation and higher albumen pH values compared to normal eggs. Based on the analysis of the presence of fatty acids, the nutritional value of double-yolked eggs was found to be slightly higher than that of single-yolked ones. Significant differences were found for some fatty acids both when analysing whole eggs and individual yolks, indicating that DY eggs could be used as a valuable source of these components. The values of the fatty acid indices showed that double-yolked eggs do not differ in quality from normal eggs, with higher amounts of n9 group acids and desirable fatty acids (DFA).

## Figures and Tables

**Table 1:** Type of double-yolked eggs.

Type	Description	Own Photos
A	The result of 2 yolks encountering one another in the infundibulum, where fertilisation occurs. Both yolks are in close contact, may have separate vitelline membranes and are enclosed in a shared layer of chalaziferous albumen.	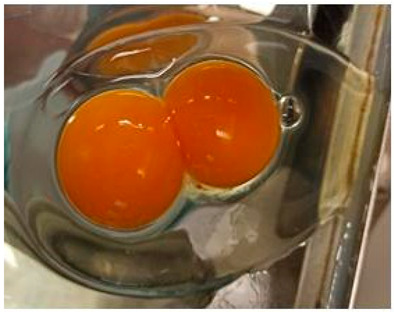
B	The yolks encounter one another in the magnum where the albumen is secreted. They have a separate chalaziferous layer of albumen, and there may be an inner layer of thin albumen in between; they share a dense albumen.	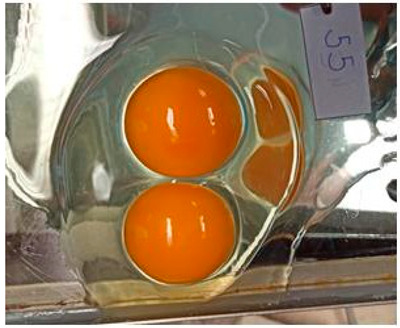
C	Yolks surrounded by separate dense albumen layers inside a shared shell membrane. This happens when the second yolk reaches the magnum while the first yolk is still in it, but the process of albumen secretion is not yet complete.	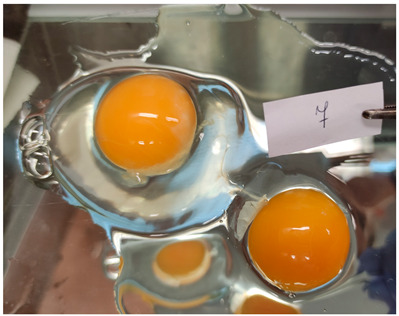
D	If the fusion of the yolks occurs at a distal part of the reproductive tract, e.g., at the isthmus or further on, a proper double egg (considered by some as the type D of DY eggs) is formed, i.e., yolks surrounded by separate albumen and shell membranes or a so-called egg-in-egg may be formed.	Not found in this study.

**Table 2:** The whole egg quality traits.

Trait		SY	DY	SEM
Shape index		80.06 *	74.53 *	0.415
Specific gravity (g/cm^3^)		1.06 *	1.07 *	0.019
Weight (g)		55.25 *	80.04 *	0.915
Proportion (%) of	Albumen	59.25 *	53.38 *	0.552
	Yolk	17.95 *	35.83 *	0.715
	Eggshell	12.65	10.28	0.133

SY—single-yolked eggs (control group), DY—double-yolked eggs, SEM—standard error of mean; *—means in the row differ significantly at *p* < 0.05.

**Table 3:** The eggshell quality traits.

Trait	SY	DY	SEM
Strength (N)	41.51	36.23	0.751
Colour (%)	41.30	41.51	0.460
Weight (g)	6.99 *	8.21 *	0.095
Cohesiveness (g/cm^2^)	103.52 *	93.78 *	1.001
Thickness (mm)	0.323	0.329	0.003
Density (g/cm^3^)	1.39	1.12	0.017

SY—single-yolked eggs (control group), DY—double-yolked eggs, SEM—standard error of mean; *—means in the row differ significantly at *p* < 0.05.

**Table 4:** The egg content quality traits.

Trait	SY	DY	SEM
Albumen			
Weight (g)	32.75 *	43.21 *	0.506
Height (mm)	8.13 *	7.52 *	0.117
Haugh units	91.11 *	80.29 *	0.841
pH	8.84 *	9.09 *	0.036
Yolk			
Weight (g)	15.29 *	28.80 *	0.112
Colour (pts)	6.95 *	6.14 *	0.093
pH	5.88	5.91	0.015

SY—single-yolked eggs (control group), DY—double-yolked eggs, SEM—standard error of mean; *—means in the row differ significantly at *p* < 0.05.

**Table 5:** The chemical composition of egg yolks.

Item (%)	SY	DY		SEM
		Yolk A	Yolk B	
Dry matter	50.41	48.37	48.33	0.435
Crude protein	15.19 ^a^	15.92 ^b^	16.24 ^b^	0.759
Crude fat	52.90	54.37	53.32	0.108
Crude ash	1.78	1.62	1.76	0.029

SY—single-yolked eggs (control group), DY—double-yolked eggs, yolk A—first of the double yolks; yolk B—second of the double yolks SEM—standard error of mean; ^a,b^—means in the row differ significantly at *p* < 0.05.

**Table 6:** Fatty acid profile of the yolks analysed.

Fatty Acid (%)	SY	DY		SEM
		Yolk A	Yolk B	
SFA				
C14:0	0.483 ^b^	0.430 ^a^	0.431 ^a^	0.006
C15:0	0.095	0.080	0.080	0.003
C16:0	27.455	26.743	27.240	0.234
C17:0	0.162	0.174	0.174	0.004
C18:0	7.097 ^a^	7.546 ^b^	7.507 ^a^	0.061
C20:0	0.023	0.027	0.025	0.001
C21:0	1.245 ^a^	1.372 ^b^	1.339 ^ab^	0.017
C24:0	0.327 ^a^	0.380 ^b^	0.337 ^ab^	0.010
MUFA				
C14:1 n5	0.165 ^b^	0.138 ^a^	0.140 ^a^	0.005
C16:1 n7	5.735 ^b^	5.048 ^a^	5.064 ^a^	0.123
C18:1 n9	38.828 ^a^	39.879 ^b^	39.851 ^b^	0.176
C20:1 n15	0.157 ^a^	0.187 ^b^	0.189 ^b^	0.005
C20:1 n9	0.105	0.108	0.113	0.003
C22:1 n9	0.055 ^b^	0.040 ^a^	0.062 ^b^	0.003
PUFA				
C18:2 n6	12.998	12.821	17.106	1.650
C18:3 n6γ	0.110	0.110	0.110	0.003
C18:3 n3α	0.413	0.387	0.387	0.007
C20:2 n6	0.147	0.154	0.151	0.002
C20:3 n6	tr	0.020	0.040	0.007
C22:2 n6	tr	0.020	0.020	0.007

SY—single-yolked eggs (control group), DY—double-yolked eggs, SFA—saturated fatty acids, MUFA—monounsaturated fatty acids, PUFA—polyunsaturated fatty acids, SEM—standard error of mean; ^a,b^—means in the row differ significantly at *p* < 0.05; tr—trace (LOD < 0.05); C14:0—myristic acid, C15:0—pentadecylic acid, C16:0—palmitic acid, C17:0—ISO-isoheptadecanoic acid, C18:0—stearic acid, C20:0—eicosanoic acid, C21:0—heneicosanoic acid, C24:0—lignoceric acid, C14:1 n5—myristoleic acid, C16:1 n7—palmitoleic acid, C18:1 n9—oleic acid, C20:1 n15—cis-5 eicosenoic acid, C20:1 n9—cetoleic acid, C22:1 n9—erucic acid, C18:2 n6—linoleic acid (LA), C18:3 n6—γ-linolenic acid (GLA), C18:3 n3—α-linolenic acid (ALA), C20:2 n6—8,11-eicosadienoic acid, C20:3 n6—dihomo-γ-linolenic acid, C22:2 n6—docosadienoic acid.

**Table 7:** Fatty acid profile of the yolks analysed.

Fatty Acid Index	SY	DY		SEM
		Yolk A	Yolk B	
SFA	36.873	36.738	36.664	0.090
MUFA	45.045	45.398	45.454	0.166
PUFA	13.668	13.457	13.423	0.220
n3	0.363	0.387	0.377	0.009
n6	13.255	13.070	13.037	0.214
n9	38.988 ^a^	40.021 ^b^	40.043 ^b^	0.168
PI	15.317	15.110	19.393	1.653
AI	0.501	0.484	0.492	0.004
TI	1.896	1.905	1.936	0.033
DFA	65.810	66.401	66.383	0.119
HSFA	27.942	27.173	27.671	0.210
H/h	1.880	1.967	2.090	0.060

SY—single-yolked eggs (control group), DY—double-yolked eggs, SFA—saturated fatty acids, MUFA—monounsaturated fatty acids, PUFA—polyunsaturated fatty acids; SEM—standard error of the mean; ^a,b^—means in the row differ significantly at *p* < 0.05; PI—peroxidizability index, AI—atherogenicity index, TI—thrombogenic index, DFA—desirable fatty acids, HFSA—hypercholesterolaemic saturated fatty acids, H/h—hypocholesterolaemic/hypercholesterolaemic ratio.

## Data Availability

The data presented in this study are available on request from the corresponding author.
